# Development of the clinical candidate PBD-C06, a humanized pGlu3-Aβ-specific antibody against Alzheimer’s disease with reduced complement activation

**DOI:** 10.1038/s41598-020-60319-5

**Published:** 2020-02-24

**Authors:** Thore Hettmann, Stephen D. Gillies, Martin Kleinschmidt, Anke Piechotta, Koki Makioka, Cynthia A. Lemere, Stephan Schilling, Jens-Ulrich Rahfeld, Inge Lues

**Affiliations:** 1 Vivoryon Therapeutics AG, Weinbergweg 22, 06120 Halle (Saale), Germany; 2 Provenance Biopharmaceuticals, 70 Bedford Rd, Carlisle, MA 01741 USA; 3 Fraunhofer Institute for Cell Therapy and Immunology, Department Molecular Drug Biochemistry and Therapy, Weinbergweg 22, 06120 Halle (Saale), Germany; 4 Ann Romney Center for Neurologic Diseases, Brigham and Women’s Hospital, Harvard Medical School, 60 Fenwood Road, Boston, MA 02115 USA

**Keywords:** Endocytosis, Antibody therapy, Drug discovery

## Abstract

In clinical trials with early Alzheimer’s patients, administration of anti-amyloid antibodies reduced amyloid deposits, suggesting that immunotherapies may be promising disease-modifying interventions against Alzheimer’s disease (AD). Specific forms of amyloid beta (Aβ) peptides, for example post-translationally modified Aβ peptides with a pyroglutamate at the N-terminus (pGlu3, pE3), are attractive antibody targets, due to pGlu3-Aβ’s neo-epitope character and its propensity to form neurotoxic oligomeric aggregates. We have generated a novel anti-pGlu3-Aβ antibody, PBD-C06, which is based on a murine precursor antibody that binds with high specificity to pGlu3-Aβ monomers, oligomers and fibrils, including mixed aggregates of unmodified Aβ and pGlu3-Aβ peptides. PBD-C06 was generated by first grafting the murine antigen binding sequences onto suitable human variable light and heavy chains. Subsequently, the humanized antibody was de-immunized and site-specific mutations were introduced to restore original target binding, to eliminate complement activation and to improve protein stability. PBD-C06 binds with the same specificity and avidity as its murine precursor antibody and elimination of C1q binding did not compromise Fcγ-receptor binding or *in vitro* phagocytosis. Thus, PBD-C06 was specifically designed to target neurotoxic aggregates and to avoid complement-mediated inflammatory responses, in order to lower the risk for vasogenic edemas in the clinic.

## Introduction

Alzheimer’s disease (AD) is a neurodegenerative disease that accounts for 60–70% of dementia patients. Progressive memory loss, and cognitive dysfunction and premature death 3–9 years after diagnosis are typical for AD^1^ and 140 million AD patients or related dementia patients are projected to require treatment and care in 2050 worldwide, generating a major socio-economic burden^2–5^. Currently, the disease is often treated symptomatically with drugs that transiently improve neuropsychiatric symptoms by antagonizing acetylcholinesterase (AChE) or N-Methyl-D-aspartic acid (NMDA) receptor alone or in combinations^6^. In contrast, disease-modifying therapies (DMTs) are new therapeutics, which aim at preventing or slowing AD progression by targeting the disease-causing mechanisms of AD^7–9^.

Most DMTs against AD in late stage clinical development either target amyloid- or tau-related pathologies. These two histopathological hallmarks are identified either as extracellular plaques of aggregated amyloid β (Aβ) peptides or as intracellular aggregation of hyperphosphorylated tau proteins in neurofibrillary tangles^10,11^. According to the amyloid hypothesis of AD^11^, proteolytic cleavage of the amyloid precursor protein (APP) results in the generation and accumulation of soluble Aβ monomers that further aggregate into soluble oligomers and fibrils of different sizes and functionalities^12^. These soluble aggregates may aggregate further into larger protofibrils and fibrils, which may be deposited as extracellular plaques or form cerebral amyloid angiopathy (CAA). Since Aβ deposits can be detected many years before pathological and behavioral AD symptoms occur and types of familial AD are caused by processing of APP and clearance of Aβ^13^, much focus has been devoted to the development of DMTs against Aβ. These include inhibitors against the proteolytic enzymes gamma-secretase and beta-secretase involved in Aβ peptide genesis. Recent failures in late stage clinical trials with some of these inhibitors however^14–16^, have questioned this therapeutic approach and the amyloid hypothesis in general^17^, although drug and clinical trial design issues, off-target effects, CNS penetration and late treatment start within the course of AD might have contributed significantly to the outcome of these clinical studies^14,16^.

The most promising DMTs against AD are based on immunotherapy^7,9^, including monoclonal antibodies that recognize specific epitopes within the Aβ peptide and preferentially bind to either Aβ monomers, oligomers or fibrils. Some of these antibodies have been engineered to avoid the induction of vasogenic edemas, which are identified as amyloid-related imaging abnormalities ARIA-Edema (ARIA-E) and ARIA-microHemorrhage/Hemosiderosis (ARIA-H)^18^. The recent controversial results of anti-Aβ antibodies in late stage clinical trials^19–22^, as well as the continued search for the right target population, ideal Aβ target epitope and antibody design point to the complexity of finding safe and efficient therapeutic antibodies against AD.

We^23,24^ and others^25,26^ have identified N-truncated and post-translationally modified Aβ peptides with a pyroglutamate at the N-terminus (pGlu3-Aβ, pE3-Aβ) as a novel therapeutic target. pGlu3-modified Aβ peptides do not have a physiological function but are increasingly found in Aβ deposits as the AD pathology progresses^27–33^. The N-terminal pGlu3-modification enhances the formation of hydrophobic Aβ oligomers that have been found to be particularly synapto/neurotoxic^34–38^. Blocking the generation of pGlu3-Aβ peptides via glutaminyl cyclase (QC) inhibitors has been shown to attenuate AD-like pathology and to rescue cognitive function in mice^39,40^. A first QC inhibitor, PQ912, has completed early clinical trial in AD patients^41,42^ and is currently entering advanced clinical development.

Targeting the pGlu3-Aβ epitope via immunotherapeutic approaches^7,43,44^ are promising therapeutic concepts, which have been substantiated by several studies in mice with AD-like pathology^23–25^. Antibodies against pGlu3-Aβ will not cross-react with amyloid precursor protein (APP) or other mature peptides in the periphery or brain, thereby avoiding peripheral antibody adsorption and off-target toxicities. We here describe PBD-C06, a highly specific monoclonal antibody against pGlu3-Aβ that has been optimized to minimize the risk for immunogenicity and ARIA-E.

## Results

### *In vitro* target binding profile

**Figure 1:**
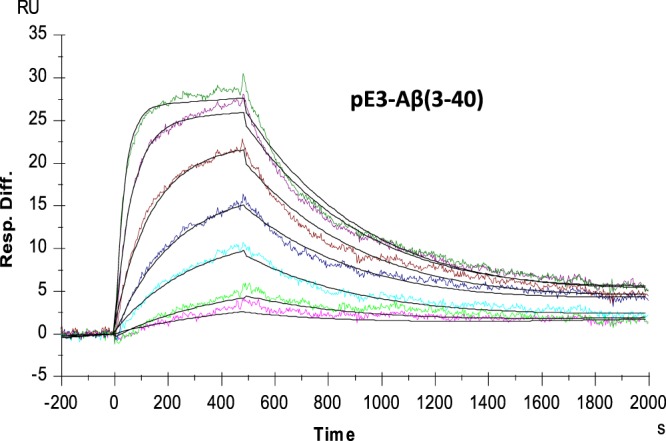
Binding of murine PBD-C06 to Aβ monomers. Sensograms of human pE3-Aβ(3–40) monomers binding to murine PBD-C06 were obtained with a Biacore 3000 SPR instrument. Binding was determined immediately after monomer preparation, using a flow rate of 30 µl/min and injecting 240 µl of 1 nM (light magenta) to 100 nM (green) of pE3-Aβ(3–40). The sensograms were recorded for 2500 sec and association and dissociation rates were obtained using the 1:1 Langmuir binding model. Resp.Diff. = Difference in Response Units.

A kinetic analysis of the interaction of monomeric pE3-Aβ peptides with the murine PBD-C06 precursor antibody by SPR revealed K_D_ constants of 7.4 nM and 6.7 nM for pE3-Aβ(3–40) and pE3-Aβ(3–18), respectively (Table 1 and Fig. 1). Dissociation constants (K_D_) of Aβ(1–40) and Aβ(3–40) peptides were about 850-fold and 100-fold higher compared to pE3-Aβ(3–40), suggesting a highly specific binding of murine PBD-C06 to pyroglutamate-modified Aβ peptides. The equilibrium (K_D_) and rate constants (k_a_ and k_d_) for pE3-Aβ(3–18) and pE3-Aβ(3–40) monomers did not differ significantly, which corroborates a previous observation that the mid and COOH-terminal regions of pGlu3-Aβ are not critical for PBD-C06 binding^45^.

**Table 1:** Murine PBD-C06 Binding to Monomeric Aβ Species.

	pE3-Aβ (3–40)	pE3-Aβ (3–18)	Aβ (1–40)	Aβ (3–40)
K_D_ (nM)	7.4	6.7	6310	796
k_a_ (s^−1^M^−1^)	2.7 × 10^5^	6.9 × 10^5^	3.9 × 10^2^	3.2 × 10^3^
k_d_ (s^−1^)	2.0 × 10^−3^	4.6 × 10^−3^	2.5 × 10^−3^	2.5 × 10^−3^

Compared to pE3-Aβ(3–40) monomers, bivalent binding of murine PBD-C06 to soluble pE3-Aβ(3–42) oligomers (200-mer) resulted in a 850-fold improved K_D_ value, whereas the binding strength did not differ significantly between pE3-Aβ oligomers (200-mer) and fibrils (Table 2). Thus, PBD-C06 forms most stable complexes with pE3-Aβ-containing soluble aggregates.

**Table 2:** Murine PBD-C06 Binding to Soluble Aβ Species.

	Monomer pE3-Aβ(3–40)	Oligomer (200-mer) pE3-Aβ(3–42)	Fibrils pE3-Aβ(3–42)
K_D_ (pM)	7400	8.7	1.0
k_a_ (s^−1^M^−1^)	2.7 × 10^5^	3.2 × 10^6^	1.3 × 10^6^
k_d_ (s^−1^)	2.0 × 10^−3^	2.8 × 10^−5^	1.4 × 10^−6^

Soluble aggregates of Aβ peptides form complex mixtures during the pathology of AD that differ in size, conformation and post-translational modifications^46^. We sought to measure the binding strength of murine PBD-C06 to various mixtures of pE3-Aβ(3–42) with Aβ(1–42) oligomers and fibrils, thus attempting to simulate murine PBD-C06 target binding *in vivo*. When equal pE3-Aβ(3–42) and Aβ(1–42) fractions were used (Table 3), the binding strength of murine PBD-C06 to these oligomeric and fibrillar mixtures did not change significantly compared to pure pE3-Aβ(3–42) aggregates. Even mixed oligomers and fibril preparations containing 5% of pE3-Aβ(3–42) were bound with low picomolar avidity, whereas oligomers consisting of 100% Aβ(1–42) interact only with nanomolar binding strength. Thus, murine PBD-C06 binds to different aggregates containing pE3-Aβ peptides with high efficiency, a prerequisite for the clearance of these aggregates *in vivo*.

**Table 3:** Murine PBD-C06 Binding (K_D_) to Aβ Mixtures.

pE3-Aβ(3–42): Aβ(1–42) (%)	100: 0	50: 50	5: 95	0: 100
Mixed Oligomers (AADL) (pM)	22.9	31.2	82.1	2020
Mixed Fibrils (pM)	1.0	1.5	34.7	1810

### Humanization and framework substitution

The complementary determining regions (CDRs) within the murine VH and VL sequences were identified by standard antibody structure modeling^47–52^. A protein BLAST search^53^ was used to identify closely related human VH and VL framework sequences. Based on 82% sequence homology, the human variable kappa sequence BAC01730.1 (Accession Number AB064102.1, family IGKV2-30*01) was selected as a suitable VL framework. For the human VH sequence, AAS85817.1 (accession number AY392875.1, family IGHV1-3*01) was chosen, having a 62% homology to the murine sequence. Subsequently, the murine CDR were grafted onto the human VH and VL genes by standard cloning techniques, thereby replacing the human CDRs. Finally, human IgG1 heavy chain constant gene and human kappa light chain constant region were fused to the framework genes creating a humanized IgG1:kappa version of PBD-C06, in which the murine CDR sequences are preserved.

**Figure 2:**
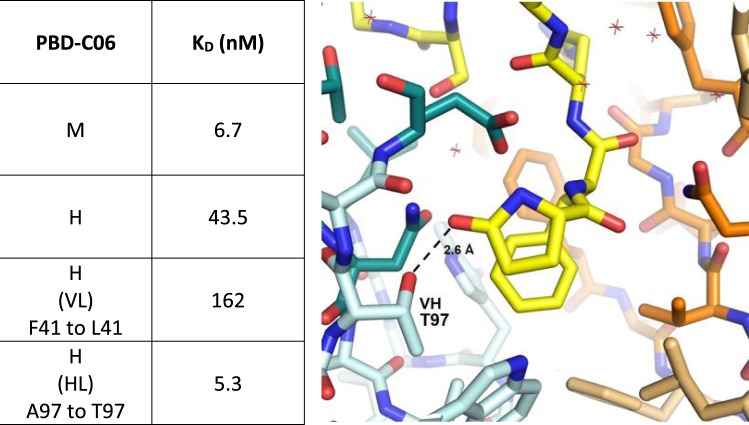
VH chain interaction with pE3-Aβ(3–12). Binding strength of murine (M), humanized (H) PBD-C06 and two designed framework mutations were determined by Biacore (left). Molecular interactions of the pyroglutamate ring at the N-terminus of pE3-Aβ(3–12) of the VL chain (yellow stick) with the hydroxyl group of T97 (cyan stick) within the murine VH chain are shown on the right as determined in^45^. The distance between T97 of the VH chain and the pyroglutamate ring of pE3-Aβ(3–12) is shown as a dashed line. Structural figures were prepared using PyMOL software (PyMOL Molecular Graphics System, version 1.5.0.3; Schrödinger).

**Figure 3:**
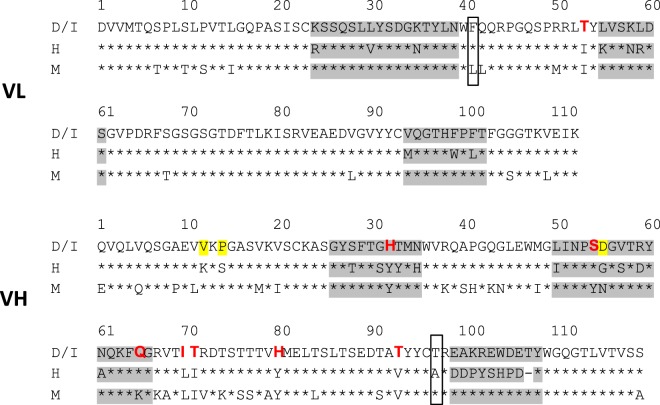
PBD-C06 sequences of VL and VH chains. Amino acid sequence including CDRs (grey), de-immunized amino acids (red) and substitutions for protein stability (yellow). D/I represents the de-immunized sequence of PBD-C06 and H and M are the human IGHV1-3*01 and murine hybridoma precursor sequences, respectively. Boxes indicate two framework regions potentially involved in antigen recognition.

Initial pE3-Aβ(3–18) binding measurements revealed a 6.5-fold reduced affinity by the humanized PBD-C06 antibody compared to its murine precursor (K_D_ = 6.7 nM vs 43.5 nM see Fig. 2). Prior structural analyses^45^, suggested that framework residues adjacent to the murine CDRs might participate in target binding. Two residues, L41 of the VL and T97 of the VH murine framework, are substituted by F41 and A97 in the humanized framework sequences, respectively (Fig. 3). Since these residues potentially participate in target binding, substitutions of the humanized residues with the original murine residues were studied. Reverting F41 within the human VL framework to the murine equivalent L41 did not improve target affinity (Fig. 2). However, substituting A97 with T97 improved the antigen affinity to 5.3 nM, thereby rescuing the humanized PBD-C06 binding strength to affinities seen with the original murine antibody.

### De-immunization

De-immunization was achieved by identifying overlapping peptides of at least 8 amino acids that bind to MHC Class II receptors with high binding strength by standard *in-silico* methods. Subsequently, critical non-germ-line alleles within each peptide were reengineered to reduce binding affinity. As shown in Fig. 3, one amino acid of the VL sequence at position 53 was identified and reengineered with a conservative substitution (I to T). The mutated VL chain was examined in whole antibody expression and target binding studies and found to have equivalent functionality to that of the original sequence (data not shown).

Within the VH chain sequence seven amino acids were identified that required substitutions. Three of these changes were within the CDR regions and four within the framework region (Fig. 3). Several amino acid substitutions at each indicated VH sequence position were first tested for protein expression individually in combination with the mutated VL chain. Once suitable substitutions were identified, combinations of all VH sequence mutations were tested for protein expression together with the mutated VL chain until an antibody with the VH and VL chain sequences depicted in Fig. 3 was generated.

**Figure 4:**
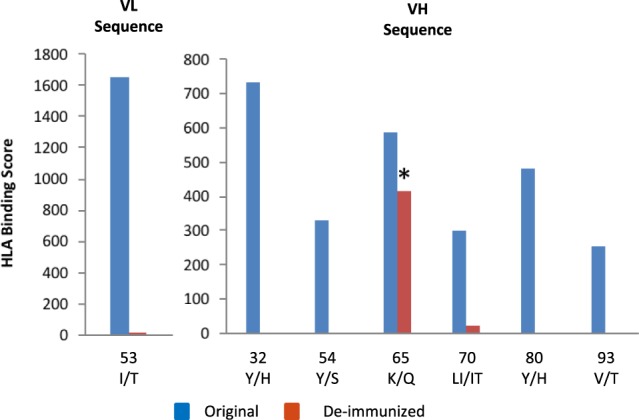
HLA binding scores of critical VL and VH peptides. In silico peptide binding to human MHC II molecules before (blue) and after de-immunization (red). *Indicates a conversion to germline amino acid.

A summary of all allelic substitutions within the VH and VL chain is presented in Fig. 4. Based on our in-silico binding analyses, the immunogenic hotspots within the VL and VH chains could be eliminated except for position 65 of the VH chain. A lysine (K) to Glutamine (Q) substitution at this position was the only choice to reduce the HLA binding strength without sacrificing substantial antigen binding affinity. This change converted the peptide to a germline sequence that should not be recognized due to central tolerance mechanisms.

### PBD-C06 expression

Upon comparison of the primary amino acid sequence against the VH regions of several antibodies with high or low expression levels, it was noted that certain residue motifs within the VH chain might influence protein stability, expression and folding of the mature antibody. For these purposes four sequence motifs were identified that may negatively influence protein production. Individual mutations and combinations thereof were introduced into antibodies containing all immunogenicity modifications and tested for protein expression in transient HEK293 cell transfections. Exchanging residues at position 12 to 14 from KKS to VKP, position 55 from N to D and position 101 from K to Q of the VH chain, respectively, resulted in expression titers several-fold higher than those obtained with the parental antibodies. Exchanging F to V at position 64 or combining changes at position 12 to 14 (KKS to VKP) with K to Q at position 101 had a negative impact on expression. The combination of the three positive mutations still resulted in improved, but not optimal expression. However this combination greatly reduced antigen binding. Thus, the exchanges at position 12 to 14 and 55 of the VH chain (Fig. 3) resulted in the highest antibody titers, while retaining full antigen binding and were included into the primary amino acid sequence of PBD-C06, to guarantee optimal expression titers of PBD-C06 during the production phase.

### Target binding of the final PBD-C06 candidate

**Figure 5:**
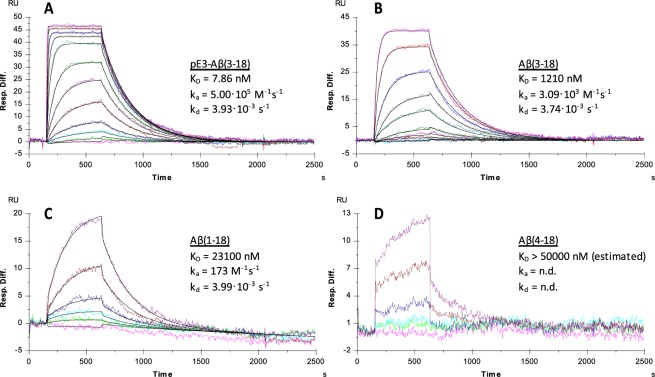
Binding of four Aβ monomers to PBD-C06. The interactions of four monomeric Aβ peptides pE3-Aβ(3–18) (**A**), Aβ(3–18) (**B**), Aβ(1–18) (**C**) and Aβ(4–18) (**D**) to PBD-C06 were analyzed with a Biacore 3000 SPR instrument. 2150 RU of PBD-C06 were captured on a Biacore CM5 Chip via an immobilized anti-human antibody. The interactions of the Aβ peptides with the captured PBD-C06 antibody were analyzed with a flow rate of 30 µl/min and injecting 240 µl of different concentrations of the peptides (pE3-Aβ(3–18): 0.2 (light magenta) – 1000 nM (light magenta – upper curve), Aβ(3–18): 10 (light cyan) – 10000 nM (light magenta), Aβ(1–18): 200 (light magenta) – 10000 nM (magenta) and Aβ(4–18): 200 (light magenta) – 10000 nM (magenta)). The sensorgrams were recorded for 2500 sec and evaluated using the 1:1 Langmuir binding model.

Based on the current results, a final antibody sequence was assembled, which contained all described modifications. This antibody, PBD-C06, is a humanized, de-immunized version of the original hybridoma clone with additional sequence modification to improve protein expression, stability and to inhibit complement activation. PBD-C06 was tested in target binding studies under the same conditions as had been done with the murine antibody (Table 1). As shown in Fig. 5, the binding strength of PBD-C06 to monomeric pE3-Aβ(3–18) is nearly identical to the murine version, as is the high specificity for the pyroglutamated Aβ(3-x) peptides. Based on these binding measurements, we assume that the amino acid modifications in the final PBD-C06 antibody did not change the target binding profile as seen with the murine precursor antibody.

### Inactivation of C1q binding

**Figure 6:**
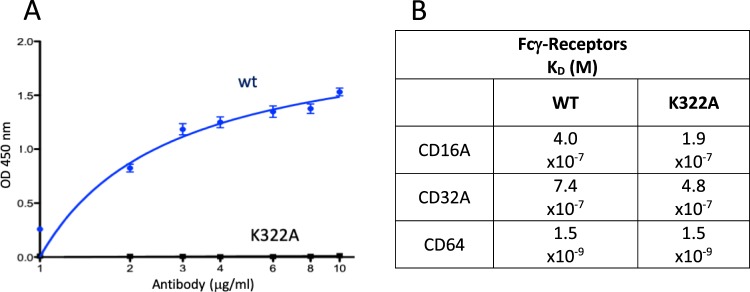
Effector function measurement of wild type and mutant PBD-C06 (K322A). (**A**) C1q binding was measured by ELISA. Plates were coated with 1 to 10 µg/ml antibody and incubated with 2 µg/ml C1q protein. For detection, a polyclonal anti-C1q antibody HRP-conjugate was used. (**B**) Fcγ-receptor binding assays were measured with a FortéBio BLItz system. Streptavidin sensors were loaded with 100 μg/ml of biotinylated CD16a, CD32a and CD64a proteins, respectively. Association and dissociation rates were measured at five different concentrations (400 μg/ml, 200 μg/ml, 100 μg/ml, 50 μg/ml and 25 μg/ml) of each antibody.

The ADCC effector function of IgG1 antibodies is involved in the phagocytotic removal of insoluble Aβ deposits, while C1q binding to the Fc domain could potentially induce neuroinflammatory processes and vasogenic edema when anti-Aβ antibodies bind to vascular amyloid^54^. In order to minimize the risk for vasogenic edema, we modified the C1q binding site within the Fc region of PBD-C06, while preserving the Fcγ-receptor binding capability. Prior work by Idusogie *et al*.^55^ suggested that a specific alanine substitution at position 322 (K to A) of human IgG1 antibodies significantly inhibited C1q binding and complement activation, while preserving ADCC functionality. C1q binding and Fcγ-receptor binding were measured with a K322A-modified PBD-C06 antibody (Fig. 6). The K322A mutation completely abolished C1q binding *in vitro*, while preserving binding to the three Fcγ-receptors CD16A, CD32A and CD64.

**Figure 7:**
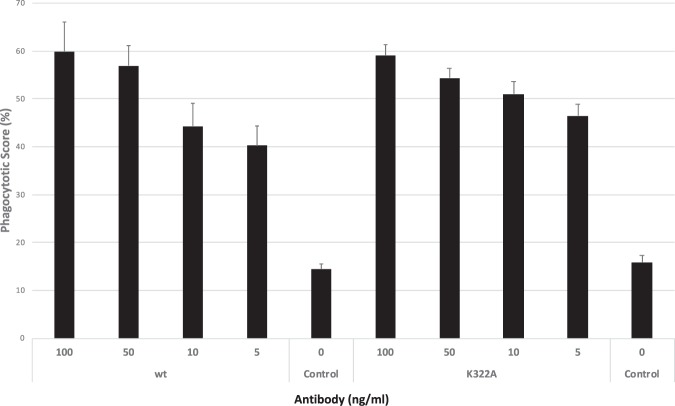
THP-1 Phagocytosis. Cellular uptake of PBD-C06 antibodies into human THP-1 cells was measured by flow cytometry. pE3-Aβ peptides coupled to fluorescent beads were first incubated with different concentrations of PBD-C06 antibodies or PBS (Control). After incubation with THP-1 cells for 24 hours, a phagocytotic score was determined as % of fluorescent cells x MFI of fluorescent cells (n = 8; +/−SD).

We next performed cellular antibody uptake experiments to ensure preservation of Fcγ-receptor mediated phagocytosis. The antibody function was assessed by means of the uptake of fluorescent beads by human THP-1 cells coated with pE3-Aβ antigen. The experiments were conducted in the presence or absence of antibody^56^ for identification of the opsonisation effect achieved by antibody binding to the antigen coated particles. As shown in Fig. 7, no significant differences in antibody mediated uptake into THP-1 cells could be observed between PBD-C06 antibodies with (K322A) or without (wt) the complement mutation. It can be concluded, that phagocytotic effector function of PBD-C06 is not impaired by the presence of the complement inactivating mutation K322A.

### pGlu3-Aβ staining by PBD-C06 variants

**Figure 8:**
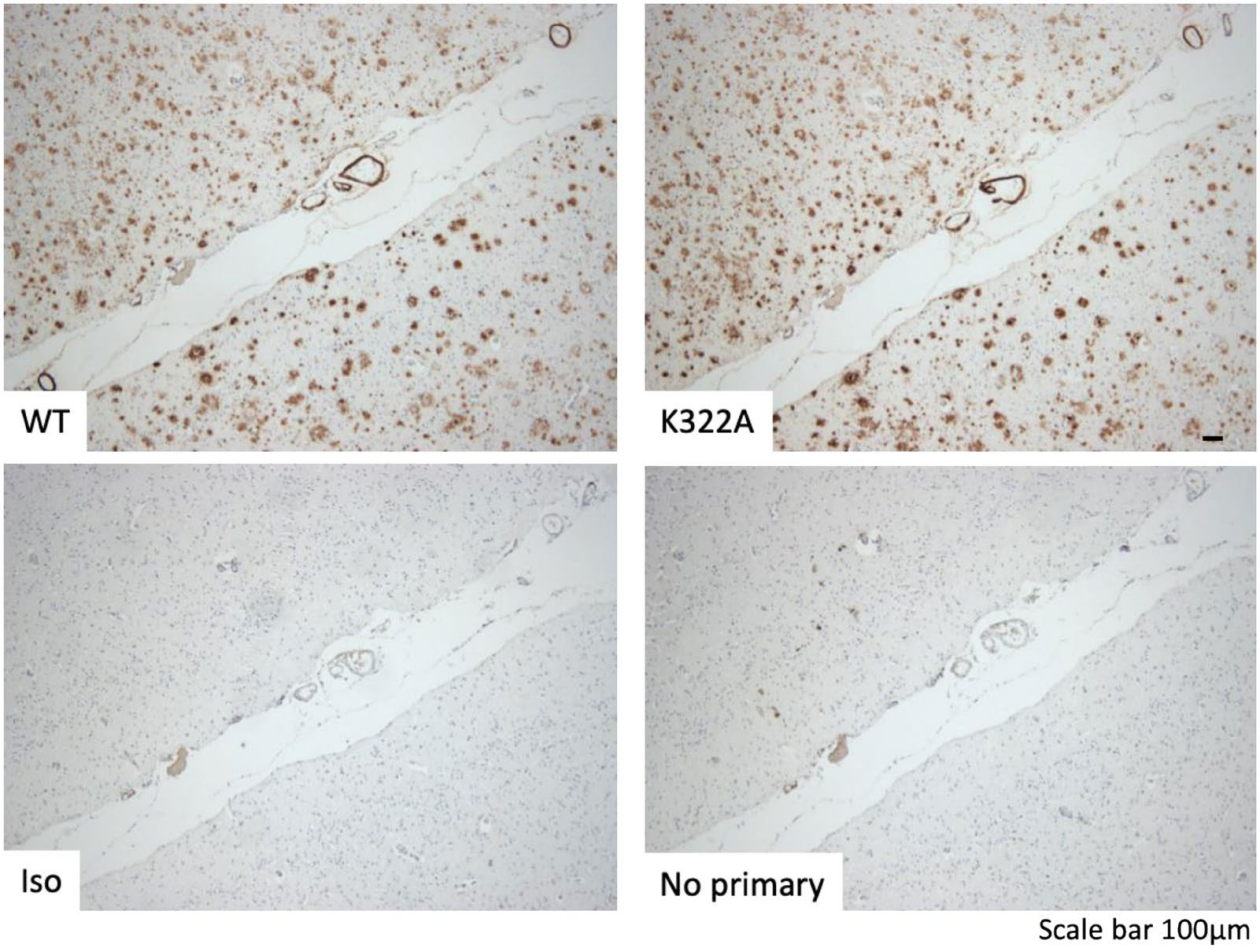
Detection of pE3-Aβ by PBD-C06 in human AD brain. Formalin-fixed (1 hour) and paraffin-embedded human AD parietal cortex sections were stained with two variants of PBD-C06. The humanized development candidate harboring the K322A mutation (K322A, top right) displayed identical staining of pE3-Aβ deposits as the parent antibody (WT, top left). Controls are provided by either using an isotype control primary human IgG1 antibody (Iso) or by omitting the primary antibody (No primary).

In a further experiment, we assessed PBD-C06’s staining specificity of pGlu3-Aβ present in amyloid deposits in the parietal cortex of human AD brain. The development candidate PBD-C06 harboring the mutation to inactivate complement demonstrated a comparable staining pattern to the wild-type antibody (Fig. 8). Thus, the humanization, de-immunization and complement inactivation modifications did not alter the *in-situ* target binding specificity.

## Discussion

In the last years, soluble aggregates of Aβ have been suggested to be the most neurotoxic Aβ species^11^, since the distribution of soluble Aβ aggregates is linked to AD pathology^11,28,57–63^. Some *in vitro* studies point to small Aβ oligomers as the most pathogenic form^63^, while others have linked neurotoxicity to larger oligomers, including protofibrils^64^. However, the soluble Aβ aggregates in the AD brain are likely a polydisperse mixture of monomers of different lengths and post-translational modifications, oligomers of various sizes and conformations, as well as larger than 70 kDa aggregates. Therefore, we have focused on generating an antibody against a neoepitope in the form of the post-translationally modified pyroglutamate ring at the N-terminus (pGlu3-Aβ) of Aβ peptides. Numerous studies point towards a crucial role of pGlu-3 Aβ in AD pathology: (i) It has been shown, that the N-termnal modification leads to rapid formation of small oligomers, which exert enhanced neurotoxicity^34^. This effect appears to have a structural basis, as mixed aggregates consisting of pGlu3-Aβ and Aβ1-42 exert also higher neurotoxicity compared to oligomers consisting only of Aβ1-42. Recent evidence suggests that this effect might be driven by different receptor interaction pattern of these oligomeric species^65^. Moreover, histopathological evidence supports a disease-driving role of pGlu3-Aβ, as the presence of the modification has been shown correlate with cognitive status of the patients and the peptide is strongly accumulating during progression of AD^27–29^. Hence, targeting of pGlu3-modified Aβ represents an approach, which is distinguished from other anti-Aβ therapies.

We here describe the development of a novel anti-pE3-Aβ therapeutic antibody, PBD-C06, intended for clinical use and treatment of AD patients. Based on crystal structure analyses, the antibody acts as a single epitope, N-terminal-specific antibody against pE3-Aβ^45^ and does not bind significantly to unmodified Aβ peptides. Thereby, the physiological functions of Aβ peptides in regulating synaptic activity or acting as an antimicrobial agent^66,67^ are preserved. The antibody binds with high specificity and avidity to pE3-Aβ oligomers and fibrils, as well as to mixed aggregates of unmodified Aβ and pE3-Aβ peptides. Aggregates with a low pE3-Aβ content are likely captured by PBD-C06 via protrusion of the N-terminal pyroglutamate ring out of these aggregates^68^. Hence, PBD-C06 has the potential to clear neurodegenerative Ab aggregates believed to be responsible for the AD pathogenesis and cognitive decline in AD patients.

The decision to select PBD-C06 as a clinical candidate was supported by a number of preclinical studies in mouse models with AD-like pathology. The murine precursor antibody inhibited pE3-Aβ(3–42) fibrillation and pE3-Aβ(3–42) oligomer-induced cell death *in vitro*^45^. The murine IgG1 version of PBD-C06 significantly reduced cerebral plaques in the absence of microhemorrhage and attenuates cognitive impairment *in vivo*^23,24^. In order to assess the influence of the effector function on efficacy, we compared the murine IgG2a and IgG1 subtypes in a therapeutic trial in APPswe/PS1dE9 mice^69^. We observed a stronger reduction of amyloid deposits and better cognitive preservation with the murine IgG2a antibody than the murine IgG1 antibody, suggesting that the effector function is associated with the therapeutic function. Therefore, we have chosen a human IgG1 isotype sequence, which is analogous to murine IgG2a, as a template for the humanization process.

We noticed that the humanization procedure resulted in a slight loss of target binding strength. The T97 side chain adjacent to the VH CDR3 region and the N-terminal carbonyl oxygen group of pE3-Aβ(3–12) are operating as hydrogen bond donor and acceptor, respectively, and are in close proximity (2.62 Å) to each other (Fig. 2). Hydrogen bond formations between these two participate in the VH interactions of the CDR region with pE3-Aβ(3–18). However, these polar interactions are absent in the presence of an alanine and its methyl side chain as present in the humanized sequence. Thus, by reverting alanine back to threonine at position 97, we reconstituted the molecular side chain interactions involved in high binding affinity.

Biological therapeutics require de-immunization in order to prevent the induction of immune response in patients. In the clinic, this immunogenicity induces high levels of anti-drug antibodies (ADA) including neutralizing antibodies^70^. For example, nearly all patients treated with the anti-pE3-Aβ antibody donanemab (LY3002813) develop ADA resulting in accelerated clearance of this antibody^71^. Seven immunogenic hotspots were identified in the humanized PBD-C06 sequence and residue substitutions were made to lower the risk of immunogenicity, while preserving protein stability and expression.

Although the murine antigen binding sequences are not fully conserved in PBD-C06, an identical pattern of antigen binding was found between PBD-C06 and its precursor (Fig. 5). Thus, despite our engineered modifications, we believe that PBD-C06 will potentially clear neurodegenerative Aβ aggregates believed to be responsible for the AD pathogenesis and cognitive decline in AD patients.

In order to address the safety concerns related to vasogenic edemas, human IgG4 or IgG2 backbones or specific Fc mutations have been chosen for antibodies such as crenezumab (Genentech), KHK6640 (Kyowa Kirin), MEDI1814 (Medimmune), ACU-193 (Acumen), ponezumab (Pfizer) or SAR228810 (Sanofi) with the intention to prevent Fcγ-receptor-mediated microglia activation and pro-inflammatory cytokine release, while binding to fibrillar Aβ deposits near cerebral vessels^18^. ARIA-E has not been observed with these antibodies, however, crenezumab and ponezumab failed in slowing cognitive decline^72,73^, suggesting that Fc-effector function might be required for treatment benefits. We have chosen the human IgG1 isotype and eliminated C1q binding by introducing a K322A substitution in the Fc region, while preserving Fcγ-receptor binding. Thus, by inhibiting C1q binding, initiation of the classical complement cascade and microglia-mediated inflammation, which may lead to vascular lesions, is inhibited^74^.

Other IgG1 antibodies which recognize conformational or unique epitopes within the Aβ peptide are aducanumab (Biogen Idec), donanemab (Eli Lilly), Ban2401 (Biogen, Eisai) and gantenerumab (Hoffman-La Roche). Most of these antibodies preferentially target larger soluble Aβ aggregates, although aducanumab also binds to insoluble Aβ fibrils. Aducanumab cleared amyloid plaques in a dose dependent manner^75^, although at the expense of inducing ARIA-E even at low doses. Donanemab and PBD-C06 are IgG1 antibodies against a neo-epitope (pGlu3-Aβ). These antibodies may have a diminished risk for acute inflammatory responses, due to the relatively low abundance of pGlu3-Aβ in amyloid aggregates and due to pGlu3-Aβ’s sequential deposition in CAA and plaques^46^. pGlu3-Aβ peptides have been described to be absent in early stages of CAA lesions^76,77^, suggesting that acute CAA-related hemorrhages may be particularly induced by anti-Aβ immunotherapy. Indeed, incidences of ARIA-E have not been reported in Phase I clinical trials with donanemab^71^. In a later update, however, ARIA-E was observed with donanemab administrations^78^. Thus, although targeting pGlu3-Aβ may in principle avoid ARIA, we decided to further reduce the risk of neuroinflammation by inhibiting C1q binding and CR3 binding to macroglia and hence, complement activation and immune cell chemotaxis.

During the submission of this manuscript, Biogen and Eisai first announced the discontinuation of two Phase III aducanumab trials (ENGAGE and EMERGE) for futility^21^. However, a later analysis revealed that the 10 mg/kg dose of aducanumab did nevertheless cause a significant reduction in decline in clinical dementia outcomes in patients of the EMERGE trial^79^. Similarly, the anti-Aβ antibody BAN2401 (Esai/Bioartic) has progressed into Phase III based on its amyloid plaque lowering and cognitive benefit profile. Although these clinical results are not discussed without reservation, they support the anti-amyloid therapy in general and ask for continued development of immunotherapeutic modalities against AD. We believe that by targeting a neo-epitope (pGlu-Aβ) and by circumventing inflammatory issues (complement inactivation) and immunogenicity (de-immunization), PBD-C06 has great potential to clear the most toxic Aβ aggregates and improve cognition in AD patients at effective doses and with acceptable safety profiles.

## Materials and Methods

### Cloning, transfection, purification and humanization

The nucleotide sequences of the humanized light chain (LC) and heavy chain (HC) were cloned into the mammalian expression vectors pcDNA3.1 and pOptiVEC-TOPO (Invitrogen), respectively. Freestyle™ CHO-S cells (Thermo Fisher Scientific, Schwerte, Germany) were transfected to transiently express the humanized antibodies according to supplier’s instructions. Murine versions of PBD-C06 with and without complement-dependent cytotoxicity (CDC) mutations, as well as experimental forms of de-immunized, humanized PBD-C06 antibodies were expressed transiently in HEK293 cells after transient transfection using Lipofectamine 2000 (Thermo Fisher Scientific) according to the manufacturer’s instructions. Antibodies were purified on a protein A agarose column (Genscript) after first removing cells by centrifugation and then filtering through a 0.22 µm filter. After loading the supernatants at a rate of 1 ml/min the column was washed with 10 volumes of PBS and 5 volumes of 50 mM Na-acetate, pH 5.5, and then eluted with 50 mM Na-acetate, pH 3.0. Antibody containing fractions were pooled, diluted and diafiltered into PBS using Amicon centrifugal filters (Millipore-Sigma, Burlington, MA).

The human variable kappa sequence BAC01730.1 (accession number AB064102.1, family IGKV2-30*01) and human VH sequence, AAS85817.1 (accession number AY392875.1, family IGHV1-3*01) were chosen for the humanization procedure.

### De-immunization

A computational algorithm^80^ was used to identify potential T-cell epitopes within the VH and VL sequences of humanized PBD-C06. Potential T-cell epitopes were predicted by scoring overlapping 9 amino acids sequences within VH and VL against the binding database of 50 HLA-DR alleles. The binding strength of each 9-mer peptide against each of the HLA-DR 50 alleles was determined and a positive binding score assigned to those peptides that displayed a binding strength above an experimentally determined threshold^81^. Subsequently, potential T-cell epitopes were removed by exchanging amino acid residues at critical positions with functional alternatives resulting in a lower binding score or conversion to a germline sequence. All conservative substitutions within the VH and VL chains were tested individually or in combination for protein expression and target binding (data not shown).

### C1q and Fcγ−Receptor binding

ELISA plates were coated with each antibody at 10, 8, 6, 4, 3, 2, 1 and 0 μg/ml in triplicate and incubated at 4 °C over-night. The plates were washed three times with PBS and then blocked with 1% BSA in PBS at 50 μl/well. C1q (Sigma-Aldrich, Taufkirchen, Germany) was added to each well at 2 μg/ml in blocking buffer and incubated for one hour at RT. The plates were then washed three times with 200 μl of PBS. Anti-C1q-HRP polyclonal antibody conjugate (Thermo Fisher Scientific) was added at a 1:250 dilution in blocking buffer (50 μl/well) for one hour. The plates were washed three times with 200 μl of PBS. 50 μl of Tetramethyl Benzidine (Thermo Fisher Scientific) was added to each well for 2 minutes. 50 μl of stop solution (1 M Sulfuric Acid) was added to each well before reading the absorbance at 450 nm.

A FortéBio BLItz system (Pall ForteBio LLC, Fremont, USA) was used to measure the antibody Fcγ-receptor binding affinities. 100 μg/ml of biotinylated CD16a, CD32a and CD64a proteins (ACROBiosystems, Cambridge, UK) were loaded onto the streptavidin sensors. Antibody association and disassociation to the receptors were measured at five different antibody concentrations (400 μg/ml, 200 μg/ml, 100 μg/ml, 50 μg/ml and 25 μg/ml). K_D_ values (and k_a_, k_d_ rates) were calculated by means of the Blitz program, provided by the manufacturer.

### Phagocytosis

A phagocytotic assay was performed according to Ackerman *et al*.^56^. In brief, THP-1 cells (DSMZ, Braunschweig, Germany) were tested for CD64 (Fcγ-RIA), CD16A (Fcγ-RIIIA) and CD32A (Fcγ-RIIA) expression. Avidin labeled fluorescent beads (FluoSpheres® NeutrAvidin®-Labeled Microspheres (1.0 μm)) were obtained from Thermo Fisher Scientific (Thermo Fisher). Biotinylated Aβ (pE3-Aβ(1–17)-PEG-Biotin) was generated at the Fraunhofer IZI (Institut fuer Zelltherapie und Immunologie, Halle, Germany). The beads were incubated with pE3-Aβ(1–17)-PEG-Biotin (500 μg/ml) over-night at 4 °C. The antigen:bead mixture was washed twice with 0.1% PBS/BSA, resuspended and transferred to a round-bottom 96 well plate at 9 × 10^5^ beads per well. Final antibody concentrations (100 to 5 ng/ml in up to 8 replicates per concentration) were incubated for 2 hours at 37 °C. 200 μl of THP-1 cells at 1.0 × 10^5^ cells/ml were added per well and left incubating over-night at 37 °C and 5% CO_2_. Cells were fixed with paraformaldehyde (4%) and analyzed with an iQue® Screener (IntelliCyt, Pasadena, USA) using the ForeCyt® 4.1 Software. After FCS/SSC gating, the fluorescent signals of whole THP-1 cells were recorded as mean fluorescent intensities (MFIs) and the cellular uptake calculated as the phagocytotic score = % of fluorescent cells x MFI of fluorescent cells.

### Generation of mixed Aβ fibrils

Aβ(1–42) and pE3-Aβ(3–42) peptides were dissolved separately in 1,1,1,3,3,3-Hexafluoro-2-propanol (HFIP) to a final concentration of 1 mM according to weighted sample and incubated over-night at RT to generate disaggregated monomers (“seedless” peptides). HFIP was evaporated over-night at RT and each remaining peptide film was dissolved in 100 mM NaOH at a concentration of 500 µM. Peptide concentrations were determined by UV spectra based on an extinction coefficient of de-protonated tyrosine at pH 13 (1629.76 M^−1^ cm^−1^ at 280 nm). Aliquots from each peptide solution were used to prepare peptide mixtures with 350 µM total Aβ concentration as follows: 100% pE3-Aβ(3–42): 350 µM pE3-Aβ(3–42); 50% pE3-Aβ(3–42): 175 µM pE3-Aβ(3–42) and 175 µM Aβ(1–42); 5% pE3-Aβ(3–42): 17.5 µM pE3-Aβ(3–42) and 332.5 µM Aβ(1–42) and 0% pE3-Aβ(3–42): 350 µM Aβ(1–42) in a total volume of 40 µl. The fibrillation process was started by diluting the mixtures with 300 mM NaCl, 25 mM Na_2_HPO_4_, 25 mM KH_2_PO_4_ and 0,01% NaN_3_, to obtain a final concentration of 50 µM. The pH was adjusted to 8.7 by adding 0.1 N HCl. The samples were incubated at 37 °C for 7 days and the fibrillation progress was monitored by a Thioflavin T (ThT) spectroscopic assay^35^. The mixed fibrils were stored at 4 °C until use in Biacore binding experiments.

### Generation of mixed Aβ oligomers

The generation of mixed oligomers consisting of pE3-Aβ(3–42) and Aβ(1–42) in different ratios was performed according to a previously described method^82^. Each peptide solution was mixed to a final concentration of 2500 µM oligomers as follows: 100% pE3-Aβ(3–42) = 2500 µM pE3-Aβ(3–42); 50% pE3-Aβ(3–42) = 1250 µM pE3-Aβ(3–42) + 1250 µM Aβ(1–42); 5% pE3-Aβ(3–42) = 125 µM pE3-Aβ(3–42) + 2375 µM Aβ(1–42); 0% pE3-Aβ(3–42) = 2500 µM Aβ(1–42) in a total volume of 15 µl. Three 5 ul aliquot from each oligomer mixture were added sequentially to separate tubes containing 735 µl Ham’s F-12 medium (Thermo Fisher Scientific) at RT and immediately vortexed before the next 5 µl aliquot was added. The final four mixed Aβ oligomer preparations were incubated at 4 °C for 18 hours and centrifuged at 13,000 rpm for 10 min at 4 °C. The soluble oligomers present in the supernatants were aliquoted into low binding microtubes (Sarstedt, Germany) and stored at −80 °C until use in Biacore binding experiments.

### SPR (Biacore) antibody binding analyses

A Biacore 3000 SPR (GE Healthcare, Freiburg, Germany) was used for all interaction analyses. For the analyses of monomeric Aβ peptides (Aβ(1–18), Aβ(3–18), pE3-Aβ(3–18), Aβ(4–18), pE3-Aβ(3–40), Aβ(1–40) and Aβ(3–40)), either anti-mouse or anti-human antibodies (Thermo Fisher Scientific) were diluted 1:40 in 10 mM sodium acetate pH 5.5 and 90 µl was injected for coupling onto a CM5 chip (GE Healthcare) at a flow rate of 10 µl/min using HBS-EP (GE Healthcare) as a running buffer. The CM5 chip surface was activated by injecting 100 µl EDC/NHS (1:1 mixture of 0.1 M N-hydroxysuccinimide and 0.4 M 1-ethyl-3(3-dimethylaminopropyl)-carbodiimide).

The determination of the binding constants was performed with BIA evaluation software 4.1.1 (GE Healthcare) by simultaneously fitting the association and dissociation phases over all recorded sensorgrams using the “1:1 Langmuir binding” model. In case of baseline drifting, the “1:1 Langmuir binding with baseline drifting” model was used.

For measuring the binding of murine and humanized PBD-C06 antibodies to multimeric and mixed Aβ aggregates, pure pE3-Aβ oligomers and fibrils were captured with pE3-Aβ-specific antibodies. Pure Aβ(1–42) aggregates and Aβ(1–42)-containing mixtures were captured with an N-terminal non-pE3-Aβ-specific antibody. Their densities differed depending on the aggregate: ~800 RU for capturing pE3-Aβ(3–42) oligomers, ~15,000 to 19,000 RU for capturing mixed oligomers and ~1,100 RU for capturing mixed fibrils. The sensorgrams were evaluated from the first injection showing an antibody binding onwards using a Single Cycle Kinetics model.

### Immunohistochemistry

Human Alzheimer’s disease brain tissues were collected postmortem at the time of autopsy, having obtained informed consent for study participation from the next of kin, and following protocols approved by the Partners Human Research Committee at Brigham and Women’s Hospital (BWH, Boston, MA). We confirm that all methods were performed in accordance with the relevant guidelines and regulations.

Human AD brain blocks were collected at autopsy and fixed in 10% neutral buffered formalin for 1–2 hours (brief fixation). Tissue blocks were processed for paraffin embedding and then sectioned at 10 μm for IHC analysis at BWH. Sections were deparaffinized in two changes of Histo-Clear (National Diagnostics, Atlanta, GA) and rehydrated in graded ethanol solutions. Endogenous peroxidase activity was quenched with 0.3% H_2_O_2_ in methanol for 10 minutes. All paraffin sections were pretreated with 88% formic acid for 5 minutes for antigen retrieval. Sections were subsequently washed with water, and incubated with 10% goat serum for 20 minutes at room temperature and primary antibody (0.3 μg/ml) overnight at 4 °C. After incubation for 30 minutes at room temperature with a biotinylated secondary antibody (Vector Laboratories, Burlingame, CA), immunoreactivity was visualized with the VectorElite horseradish peroxidase ABC kit (Vector Laboratories) and DAB (Sigma-Aldrich) as chromogen.

## Data Availability

The datasets used and/or analysed during the current study are available from the corresponding author on reasonable request.
